# Increased Soluble CrkL in Serum of Breast Cancer Patients Is Associated with Advanced Disease

**DOI:** 10.3390/cancers11070961

**Published:** 2019-07-09

**Authors:** Srimeenakshi Srinivasan, Biana Godin

**Affiliations:** 1 Department of Nanomedicine, Houston Methodist Research Institute, Houston, TX 77030, USA; 2 Department of Obstetrics and Gynecology, Houston Methodist Hospital, Houston, TX 77030, USA

**Keywords:** CrkL, soluble fraction, blood serum levels, breast cancer

## Abstract

Over-expression of Crk-like protein (CrkL), an intracellular adaptor protein, in breast cancer biopsies has been linked to poor prognosis. CrkL can be secreted from cancer cells binding to β1 integrin on the cell membrane. In this study, we evaluated, for the first time, the levels of soluble CrkL in serum of breast cancer patients. Expression of CrkL and secreted fractions from human breast cancer cell lines and clinical patient samples were assessed by immunohistochemistry and Enzyme Linked Immuno-Sorbent Assay (ELISA). CrkL levels in tissues and sera of patients with different disease stages were compared and statistically analyzed by Chi-square test and Student’s *t*-test. Culture media from human breast cancer cell lines SUM159, MDA-MB231, and MCF7 showed over a 21-, 15-, and 11-fold higher concentration of soluble CrkL as compared to normal breast epithelium cell line MCF10A. Expression of CrkL was elevated in 85% of breast tumor tissue sections. Serum levels of CrkL were significantly higher in breast cancer patients than in healthy donors. All patients with metastatic disease had significantly elevated concentration of soluble CrkL in the serum with on average three-fold increase from the baseline. The data suggest that soluble fraction of CrkL can be further evaluated as a serum biomarker for advanced disease in breast cancer patients.

## 1. Introduction

Initiation and progression of many ailments causes changes in the level of expression of proteins involved in the pathologic course of the disease [1]. Proteins that are over-expressed on the cancer cells and in the tumor microenvironment (TME), as well as proteins secreted from the tumors and detected in the patient’s fluids, can be easily collected and, thus, have been and are being used in the clinic as markers for tumor prognosis and diagnosis [2]. Clinically used soluble markers for cancer diagnosis include mesothelin in malignant pleural mesothelioma [3], colony stimulating factor-1 (CSF-1) [4] for endometrial carcinoma, prostate specific antigen (PSA) for prostate cancer [5], and carcinoembryonic antigen, CA125, for ovarian cancer [6]. In breast cancer, a number of plasma biomarkers, such as vascular endothelial growth factor (VEGF) and carcinoembryonic antigens, CA 15.3 and CA 27.59, were found to have a prognostic value [7]. Today, the CA 15.3 marker is the most frequently used in the evaluation of breast tumor prognosis, together with diagnostic imaging for monitoring advanced disease stages [8]. While the knowledge about existing biomarkers is growing, it is important to find new possible biomarkers that can complement the existing panel for providing a better disease diagnosis and prognosis.

Crk-like protein (CrkL) is a protein from the Crk (CT10 Regulator of Kinase) protein family, which was initially discovered in neutrophils of patients with chronic myeloid leukemia (CML) as an intracellular adaptor protein [9]. Proteins from the Crk family have been linked to various intracellular signaling pathways involved in cancer cell migration, metastatic invasion, and survival [10]. Over-expression of CrkL in the tumor tissues has been reported in the numerous solid tumors, including prostate [11], gastric [12], hepatocellular carcinoma [13], lung [14], and breast cancers [15,16]. These reports demonstrated that the high levels of CrkL in the tumor tissue were linked with more aggressive tumors characterized by poor prognosis and reduced survival. In the work by Fathers et al. [15], the authors show that Crk proteins have a significant role in regulating malignant basal breast disease. Proteins’ location in various intra- and extracellular compartments frequently dictates their function [17]. Intracellularly located CrkL binds to tyrosine kinase phosphorylated scaffold proteins, such as C3G, paxillin, and p130Cas, thus being involved in tumor cell adhesion and migration through integrin mediated signaling. Additionally, intracellular CrkL can regulate gene transcription in the nucleus, acting as a nuclear adaptor protein for Signal transducer and activator of transcription 5 (Stat5) [18]. Although the main body of the published work is focused on the intracellular functions of CrkL, Mintz et al. have shown that there is a membrane-bound fraction of unphosphorylated CrkL, which is originated from the extracellular secretion of the protein by ABC transporters or during the apoptotic/necrotic cell death [11]. It was suggested that the secreted fraction of CrkL binds to the plexin–semaphorin–integrin (PSI) domain of β1 integrin, thus activating the mitogen-activated protein (MAP) kinase pathway, leading to nuclear transcription, and, as a result, enhancing cell division and migration [19].

The main objective of the present work was to evaluate the possibility of detecting secreted CrkL as a soluble biomarker in breast cancers, as well as to correlate serum levels of CrkL to various clinical parameters. For this purpose, we evaluated membrane-bound and secreted fractions of CrkL in human cancer cell lines and clinical tissue and blood serum samples from breast cancer patients with early and advanced disease, as well as healthy donors.

## 2. Results

### 2.1. CrkL Membranal Fraction In Vitro in Human Breast Cancer Cells

**Figure 1:**
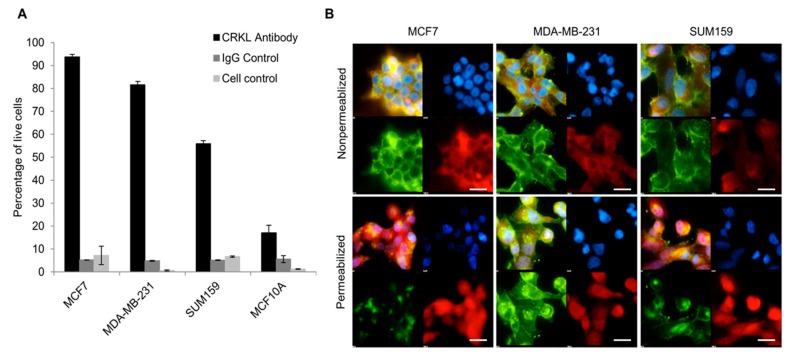
Intracellular and membranal localization of Crk-like protein (CrkL) in vitro in breast cancer cells. (**A**) Flow cytometry on non-permeabilized cells showing surface fraction of CrkL in breast cancer cells (MCF7, MDA-MB-231, and SUM159) versus normal breast epithelium cells (MCF10A), the data is presented as percentage of fluorescently labeled cells from all live cells, *n* = 6; *p* < 0.001 comparing the percentage of all the evaluated breast cancer cells with membranal fraction of CrkL versus MCF10A cells; (**B**) immunofluorescence images of human breast cancer monolayers showing the difference in the staining pattern of CrkL (**red**) in permeabilized (**bottom row**) and non-permeabilized (**top row**) cells. The cells are counterstained with wheat germ agglutinin 488 for the cell membrane (**green**) and 4′,6-diamidino-2-phenylindole (DAPI) for the nucleus (**blue**). (Scale bar = 20 µm).

Previous work has shown that CrkL, which is an intracellular protein, can be secreted from cancer cells in vitro and bind to the cell membrane through interaction with β1 integrin. To assess the secreted fraction of CrkL bound to the cell membrane (membranal fraction), a flow cytometry analysis was conducted on three non-permeabilized breast cancer cell lines, as compared to the normal breast epithelium cell line. Different cell lines exhibited various percentages of cells with surface-bound CrkL. Three- to five-fold higher percentages of staining were observed in breast tumor cell lines, namely MCF7, MDA-MB-231, and SUM 159, as compared to MCF10A cells originated from normal mammary epithelium. As an example, surface bound fraction of CrkL was detected in 93.7 ± 1.2%, 55.9 ± 1.2%, and 17 ± 3.2% of MCF7, SUM159, and MCF10A cells, respectively (Figure 1A).

Further, to confirm the membranal fraction of CrkL, breast cancer cells were immunostained and analyzed by fluorescent microscopy. In the non-permeabilized cells, there was no co-localization of CrkL antibody with a nuclear probe (4′,6-diamidino-2-phenylindole, DAPI), thus pointing towards that the observed staining is the membrane-bound fraction of CrkL. On the contrary, after the membrane of the cells was permeabilized, a significant portion of the fluorescent signal was associated with the cell cytoplasm and the nuclei since, as anticipated, an important fraction of the protein is located intracellularly (Figure 1B).

### 2.2. Soluble Fraction of CrkL Excreted in Breast Cancer Cells In Vitro

**Figure 2:**
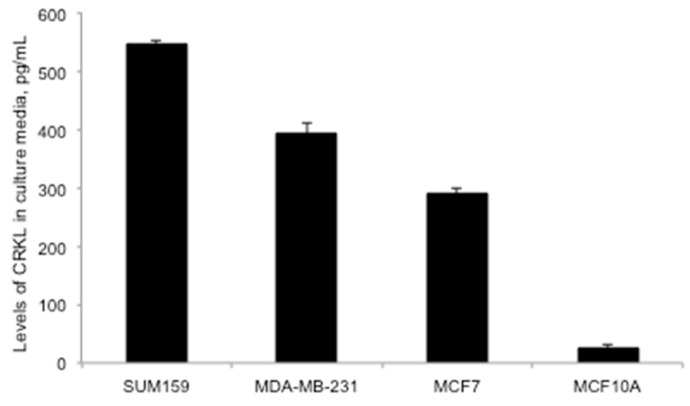
Assessment of secreted Crk-like protein (CrkL) levels in vitro in cancer media collected from breast cancer cells (MCF7, MDA-MB-231, and SUM159) versus normal breast epithelium cells (MCF10A), the data analyzed by Enzyme Linked Immuno-Sorbent Assay (ELISA), *n* = 5–6; *p* < 0.001 comparing levels in all the evaluated breast cancer cells with levels of soluble CrkL in the cell media from MCF10A cells.

Further, it was important to study the soluble fraction of CrkL excreted from the breast cancer cells in vitro and not bound to cell membrane. Conditioned media (CM) from cell culture supernatants were analyzed by Enzyme Linked Immuno-Sorbent Assay (ELISA) for secreted CrkL. CM from human breast cancer cell lines SUM159, MDA-MB231, and MCF7 showed over a 21-, 15-, and 11-fold higher concentration of soluble CrkL than CM from normal breast epithelial cell line MCF10A (545 ± 7, 393 ± 18, 289 ± 10 vs. 26 ± 5pg/mL, respectively) (Figure 2).

### 2.3. CrkL Expression in Clinical Tumor Biopsies

**Figure 3:**
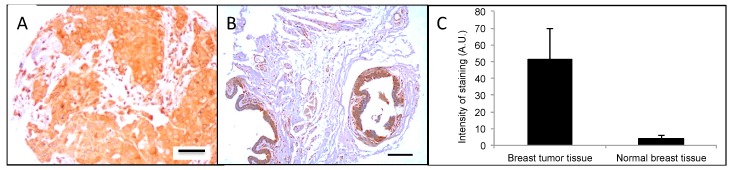
CrkL expression in human breast cancer microarray. (**A**) Immunohistochemistry for Crk-like protein (CrkL) in breast cancer tissue microarray (TMA) showing strong signal throughout the tissue. CrkL staining can be detected both in the cytoplasm (**bottom left**) and plasma membrane (**bottom middle**) in many tumors. In some tumors the CrkL is also found in the extracellular matrix. (**B**) Normal breast tissue showing positive staining in the ductal epithelium only. (Scale bar 4× magnification = 500 µm, 40× magnification = 12.5 µm). (**C**) Quantitation of intensity of staining through image analysis in tumor cores and normal breast tissues (excluding ductal regions), *p* < 0.001.

Next, CrkL expression in clinical breast cancer specimens was evaluated. For this purpose, a paraffin-embedded cancer progression tissue microarray (TMA), containing 94 tumor cores, was immunostained for CrkL. The data is presented in Figure 3 and Supplementary Figure S1. As shown in Figure 3B, normal breast tissue samples exhibited a significant CrkL signal only in the ductal epithelium. For this reason, en course of the pathological analysis of the tumor cores, the regions of ductal epithelium were excluded. The majority of the clinical samples were from Caucasians (>90%), with 27 and 46 patients having estrogen receptor (ER) negative and progesterone receptor (PR) negative breast cancer, respectively. The tumor diameter varied from 1 to 6.5 cm, with the average size being 2.6 cm.

Breast tumor biopsies varied in staining patterns for CrkL expression. Some biopsies exhibited more pronounced nuclear staining, while in others the staining intensity was increased in cytoplasmic or extracellular or microenvironmental regions. In total, 85% of the clinical breast tumor biopsies stained positive for CrkL. As presented in Figure 3C, when compared to uninvolved tissue, a 12-fold increase in the CrkL staining intensity was detected in the breast tumor biopsies (3.9 ± 1.9 vs. 48.6 ± 22.1 arbitrary units (A.U.)/pixel, respectively). The correlation of the intensity of CrkL staining and the clinicopathological factors in breast cancer tumor biopsies is summarized in Table 1. It is noteworthy that the PR and ER status had no effect on the CrkL expression in the breast tissue samples. Unfortunately, no information about the human epidermal growth factor receptor 2 (HER2) status was provided for the de-identified breast cancer tissue biopsies in the TMA.

### 2.4. CrkL Soluble Fraction in Serum from Breast Cancer Patients

**Figure 4:**
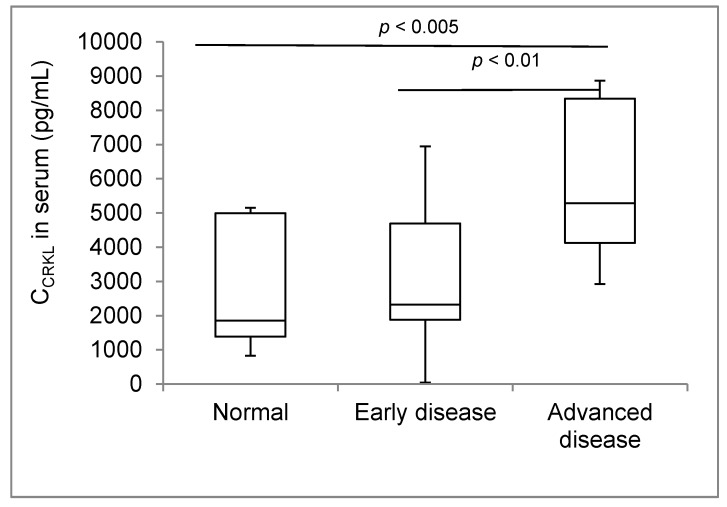
Assessment of secreted Crk-like protein (CrkL) levels in breast cancer patients’ sera. The CrkL concentration (C_CrkL_) was measured in the sera from breast cancer patients using Enzyme Linked Immuno-Sorbent Assay (ELISA).

ELISA was used to assess the soluble CrkL in sera collected from 29 breast tumor patients and 10 healthy donors. Table 2 presents the clinical data of the individual patients and the levels of CrkL in their sera, and Figure 4 shows the summary of the results based in comparing between healthy donors, patients with early disease and patients with the advanced (Stage 3 and 4) disease. The median CrkL levels in the sera of breast cancer patients were more than twice higher than in healthy individuals (4400 vs. 1860 pg/mL, respectively, *p* < 0.05). In patients with an advanced breast cancer, serum levels of soluble CrkL were 2.8-fold higher (5300 pg/mL, *p* < 0.005), as compared to the healthy donors (Figure 4). The median value of CrkL concentration in the patients’ sera was higher, as compared to healthy participants, in 42% of patients with early disease (Stage 1 and 2), 93% of patients with advanced disease (Stage 3 and 4), and in 100% of patients with metastatic disease. The sera from patients who had not undergone any treatment (*n* = 4) contained higher levels of soluble CrkL than those patients who were undergoing treatment (5800 vs. 3600pg/mL, respectively, *p* = 0.13).

There was no data on the breast cancer sub-type in the description of the de-identified serum samples from patients acquired from the bio-repository. Although in the TMA there were no differences between the CrkL staining and the PR(−) versus PR(+) and ER(−) versus ER(+) status (*p* = 0.27 and 0.11, respectively), following studies will have to examine the relationship between the breast cancer subtype and the levels of the soluble CrkL in the patients’ sera.

## 3. Discussion

The vast majority of biomarkers used in the clinic for assessing breast cancer cases are proteins that are expressed by the breast cancer cells and can be evaluated following tumor resection or biopsy in histological specimens. Presence of these biomarkers is important for disease diagnosis, evaluation of the therapeutic options, and is frequently correlated to disease prognosis [7], particularly in the early disease stages. While these biomarkers are very important and can lead to development of new therapies, as in the case of trastuzumab [20], they can be analyzed only after an invasive procedure, such as biopsy or surgical resection. This obstacle can be overcome by finding soluble biomarkers that can be detected in the body fluids. In the current work, we hypothesized that CrkL, generally considered as a protein overexpressed intracellularly in breast cancers, when secreted from the tumor cells, can possibly also serve as a serum biomarker for breast tumors. For this purpose, we evaluated CrkL levels in human breast cancer cell line media in vitro, clinical breast cancer tissue and serum samples from breast cancer patients.

**Figure 5:**
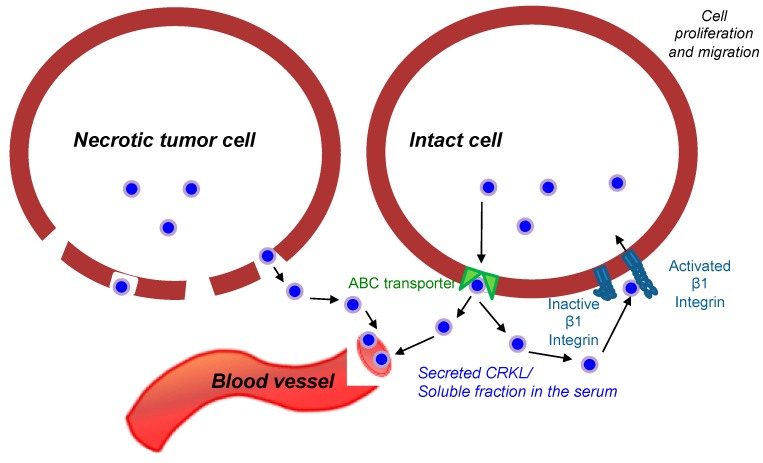
Schematic presentation of the proposed mechanisms of the presence of membranal and soluble Crk-like protein (CrkL) fractions in aggressive breast carcinoma (based on the data in Reference [11,19]).

As mentioned above, CrkL does not have transmembrane motifs and the membrane-bound fraction of the protein—bound to PSI domain of the β1 integrin—can originate only from the fraction excreted extracellularly. Previous studies have examined the presence of extracellular fraction of CrkL in the TME [11]. As summarized in Figure 5, it was proposed that unphosphorylated intracellular CrkL fraction is secreted from the tumor cells into the TME either through an active transport mechanism or as a result of cell death into the microenvironment. Further, Src homology 3 (SH3) domains bind specifically to the β1 integrin PSI domain on the membrane of the tumor and TME cells, inducing a conformational change of β1 integrin to its extended, active form, and, as a result, triggering the phosphorylation of proteins in the associated molecular pathways [11]. The relationship between the tumor invasiveness and the expression of extracellular CrkL, including the fraction that binds to the PSI domain of β1 integrin on the membrane, as well as the soluble excreted fraction that is identified in the blood sera, can be related to the effect on tumor migration and proliferation caused by activation of the above pathways [19]. In agreement with these findings, we detected an increased surface-bound fraction of CrkL in various breast cancer cell lines (Figure 1) when compared to non-cancerous epithelial cells. Immunofluorescence staining of breast cancer cells revealed CrkL both on the cell surface and in the intracellular compartments (Figure 1b). The levels of CrkL in the breast cancer cell media were significantly higher than in the normal breast epithelium cell media (Figure 2).

Similar to in vitro studies in breast tumor cells, high expression of CrkL was detected in clinical breast tumor tissue (Figure 3). While immunohistochemical staining cannot differentiate between the membrane-bound fraction of CrkL and the intracellular protein, it is in accordance with the data published by others confirming high levels of CrkL in tissues from numerous tumors, such as lung [14], gastric [12], and head and neck cancers [21]. Furthermore, a study by Zhao et al. has demonstrated over-expression of CrkL in 37% of the breast tumors. In this study, CrkL expression levels in the tumor tissue were associated with breast cancer growth and progression [16]. Our data provide further evidence that expression of CrkL in the breast tumors can serve as a potential tissue biomarker.

CrkL has been linked to several molecular pathways related to tumor invasiveness in a number of solid tumors [21]. As an example, Liu et al, using an in situ proximity ligation assay to identify and quantify 67 endogenous protein-protein interactions among 21 interlinked pathways, found that CrkL, involved in extracellular-signal-regulated kinase (Erk) pathway, can be a prognostic marker in hepatocellular carcinoma that strongly correlates with disease-free and overall survival [13]. It has been shown that increased levels of CrkL protein correlate with cancer progression in various cancers [22]. As an example, in human endometrial carcinoma, CrkL protein was overexpressed in 50.5% (44/87) of tumors, inhibiting cell apoptosis, upregulating the expression of cyclin D1, cyclin E, B cell lymphoma (Bcl)-2, and surviving, thus contributing to a more aggressive phenotype and resistance to apoptosis [23]. Similar expression patterns were found in invasive ductal breast carcinoma (IDC), where CrkL TME overexpression in 37% of the IDC breast tissue samples correlated has been found significantly correlate with advanced p-tumor-node-metastasis stage and tumor metastasis [16]. In vitro studies revealed that in breast cancer CrkL plays a regulatory role in the stromal cell-derived factor 1 (SDF-1), induced Erk1/2, and phosphatidylinositol 3-kinase/Protein Kinase B (PI3K/Akt) pathways, which are directly linked to the invasion and migration of breast cancer cells [24].

While the overexpression of CrkL in the tissue has been previously linked to breast cancer progression, we believe that this is the first report to evaluate the soluble fractions of CrkL in cell culture media and blood sera from breast cancer patients. The results show significantly higher levels of soluble CrkL in sera from breast tumor patients as compared to healthy donors, suggesting the potential use of CrkL as a soluble biomarker. CA 15-3, the soluble breast cancer biomarker most frequently evaluated in the clinic, is being used to assess diseases prognosis, monitor therapeutic responses, or to detect early relapse [25]. Nevertheless, CA 15-3 levels are significantly increased in only 3% with early stage breast cancer and less than 70% of those with advanced disease [26]. In the current work, elevated serum levels of CrkL were found in more than 40% of patients with localized or early stage disease and in all patients with advanced metastatic breast cancer. Additionally, among the patients whose sera were analyzed in our study, four had not undergone therapy and had CrkL serum levels 1.6-fold higher on average than the treated patients (*n* = 25). These data suggest that CrkL levels might also be useful in monitoring the therapeutic outcomes in breast cancer and require further evaluation in larger patient cohorts and progressive clinical studies.

There are multiple signaling pathways that can affect the expression and functionality of intracellular CrkL. The triggers of these pathways have been summarized previously (see Table 1 in Reference [27]) and include various factors at levels that change in the TME: cytokines (e.g., interferons, interleukins, Granulocyte-macrophage colony-stimulating factor (GM-CSF)), growth factors (Vascular Endothelial Growth Factor (VEGF), Fibroblast Growth Factor (FGF), Insulin-like Growth Factor (IGF), Epidermal Growth Factor (EGF)), reactive oxygen species, and others. It is unknown and hard to hypothesize about the role of the individual factors—or alternative pathways—on the extracellular release of CrkL. While our data show a link between the stage of the breast tumor and the levels of soluble CrkL in the patients’ sera, further studies will be required to assess the specific triggers of the extracellularly excreted CrkL.

## 4. Materials and Methods

### 4.1. Cell Culture

Three types of human breast cancer cell lines, MCF7, MDA-MB-231, and SUM159, were chosen and maintained in Dulbecco’s Modified Eagles Medium (DMEM), supplemented with 10% fetal bovine serum (FBS, Life Technologies^TM^, NY, USA) and 1% penicillin (100 units/mL) and streptomycin (100 μg/mL). The human breast epithelial cell line (MCF10A) to represent normal epithelium was maintained in DMEM/F12 media supplemented with 5% horse serum, 20 ng/mL epidermal growth factor (EGF, PeproTech, NJ, USA), 500 μg/mL hydrocortisone (Sigma-Aldrich Co., MO, USA), 100 ng/mL cholera toxin (Sigma-Aldrich Co., MO, USA), 10 μg/mL insulin (Sigma-Aldrich Co., MO, USA), and 1% penicillin and streptomycin.

### 4.2. Evaluation of Surface Bound CrkL by Flow Cytometry

Flow cytometry analysis was used to assess the surface fraction of CrkL. For this purpose, the cells were grown in a 6-well plate. After reaching 80% confluency, the cells were harvested, incubated for 45 min with CrkL antibody (SantaCruz Biotechnology, TX, USA, catalog #SC-9005) and further for 1 h with allophycocyanin (APC) labeled secondary antibody (SantaCruz Biotechnology, TX, USA, catalog #SC-3846), washed three times in PBS, and further re-suspended in PBS for the analysis by flow cytometry. As a control, cells incubated only with the APC-labeled secondary antibody were used. The flow cytometry analysis was performed using BD FACS Fortessa analyzer (BD Biosciences, CA, USA) at 561 nm excitation laser. At least 20 thousand cells were assessed in each sample. The data analysis focused only on live cells using electronic gate to identify the APC positive events using BD FACSDiva^TM^ software (BD Biosciences, CA, USA). All the experiments were repeated three times.

### 4.3. Collection of Conditioned Media (CM) for Evaluation of Secreted CrkL

To evaluate the levels of CrkL secreted from the breast cancer cells in culture, the cells were seeded at 50% overnight, followed by washing with PBS and 24 h incubation with serum-free media. At the end of the incubation time, CM were collected, centrifuged at 5000 rpm for 10 min, and the supernatant was stored at −80 °C until the analysis by ELISA.

### 4.4. Patient Samples

For the analysis of CrkL expression in the clinical tissues from breast cancer patients, progressive breast cancer TMAs were purchased from the Cancer Diagnosis Program (CDP Breast TMA/L-0284, National Cancer Institute). A total of 94, formalin-fixed and paraffin-embedded, breast tumor tissue blocks containing samples from different stages of disease progression classified using TNM (AJCC, American Joint Committee on Cancer, VI edition) with T1, T2, T3, and T4 were assessed. Normal breast tissues (*n* = 23) removed during surgery along with the tumorous tissue were used as controls. Although the samples were deidentified, patient-related clinical data, including the race, age, the stage of the diseases, tumor size, nodal involvement, metastatic status, and PR and ER status, was provided.

Independently of TMAs, twenty-nine breast cancer patients’ sera and nine healthy donor serum samples were purchased from ProMedDx, LLC (Norton, Massachusetts, USA) and tested for soluble CrkL fraction by ELISA. These samples were not from the same patients as the TMAs, although as in the TMAs, the serum samples were from patients with different stages of disease progression, where “early diseases” is defined as Stage 1 and 2 and “Advanced diseases” is defined as Stage 3 and 4, as classified by the Cancer Diagnosis Program using TNM staging.

Biopsies from patients taken for the TMA were approved by the Institutional Review Board (IRB) at the National Cancer Institute. Informed consent was not required for these samples. Collection and use of the patient serum for this study was approved by the IRB at Promeddx, LLC. Informed consent was obtained or implied by return of questionnaires at the physician’s site.

### 4.5. Assessment of Cellular Localization of CrkL In Vitro Using Immunofluorescence

For in vitro analysis of the CrkL levels in the breast cancer cells by immunofluorescence, the cells were cultured in eight-chamber slides (BD Biosciences, CA, USA) at 80% confluency and allowed to attach overnight. The next day, the cells were fixed with 4% paraformaldehyde (PFA, Thermo Scientific, IL, USA), and the slides were randomly divided into two groups. In the first group the cells were permeabilized with 0.2% Triton^TM^ X-100 (Sigma-Aldrich Co., MO, USA) for 5 min and washed extensively. In the second group, to assess the fraction of CrkL bound to the cell membrane, the permeabilization step was not performed. Both permeabilized and non-permeabilized cells were further incubated with CrkL antibody (Santa Cruz Biotechnology, Inc., TX, USA, catalogue #SC-9005) at 1:500 dilution for 1 h at room temperature, washed, and then incubated with Dylight 594-conjugated goat anti-rabbit secondary antibody (Thermo Scientific, IL, USA, catalogue #35560) for 45 min. After the cells were washed, they were fixed again with 4% PFA, mounted with Prolong gold containing 4′,6-diamidino-2-phenylindole, DAPI (Life Technologies^TM^, NY, USA), and the immunofluorescence was detected using a Nikon fluorescence microscope (Nikon Instruments Inc., NY, USA). All the experiments were repeated three times.

### 4.6. Enzyme Linked Immuno-Sorbent Assay (ELISA)

ELISA was used for the analysis of the concentration of soluble CrkL in vitro in cell culture media and in sera from breast cancer patients and healthy donors. A commercially available kit purchased from Cloud-Clone Corp. (TX, USA) was used in accordance with the manufacturer’s guidelines. The standard curve based on the known concentration of CrkL was used to assess the concentration of the protein in pg/mL with a detection limit of 53 pg/mL.

### 4.7. Evaluation of CrkL Expression in Tumor Samples Using Immunohistochemistry

For evaluation of CrkL expression patterns in the clinical breast cancer tissues, immunohistochemical staining was performed on paraffin-embedded tumor sections (TMA). For the staining, the tissues were deparaffinized and further blocked for 10 min in peroxidase, followed by 10 min of 2.5% horse serum (Life Technologies, NY, USA). Next, the slides were reacted with rabbit anti-CrkL antibody (1:100) for 30 min; washed three times; incubated for 15 min with horse anti-rabbit IgG; reacted for 4 min with diaminobenzidine; rinsed; counter-stained with hematoxylin; mounted; and imaged. For the analysis, 10× and 40× images were captured using a Nikon microscope and the intensity of staining was determined by image analysis software, NIS elements (Nikon Instruments Inc., NY, USA).

### 4.8. Statistical Analysis

CrkL levels on the cell surface and in the cell culture media were analyzed by Analysis of variance (ANOVA). CrkL levels in tissues and serum between patients with different disease stages were compared and statistically analyzed by Chi-square test and Student’s *t*-test. *p* < 0.05 were considered statistically significant.

## 5. Conclusions

In conclusion, in this study, we have shown that high levels of secreted CrkL can be detected in the serum of breast cancer patients with advanced disease. To the best of our knowledge, this is the first report about the possibility to detect soluble fractions of CrkL in body fluids from cancer patients. The results of our study suggest that CrkL can be proposed as a soluble serum biomarker in breast cancer patients, especially in the advanced disease stages. This data should be further verified in prospective clinical trials.

## Figures and Tables

**Table 1:** Relationship between CrkL expression and pathological parameters in clinical breast tumor biopsies.

Clinical Parameters	Negative		Positive		*p*-value
	*n*	%	*n*	%	
**Age**					
<60 years (***n*** = 51)	10	19.6	41	80.4	0.86
>60 years (***n*** = 43)	9	20.9	34	79.1	
**T stage**					
T1 (***n*** = 43)	7	16.2	36	83.8	0.001
T2 (***n*** = 40)	8	20	32	80	
T3 (***n*** = 3)	1	33.3	2	66.7	
T4 (***n*** = 8)	3	37.5	5	62.5	
**Nodal involvement**					
Negative (***n*** = 33)	3	9	30	91	0.007
Positive (***n*** = 45)	11	24.4	34	75.6	
**Metastatic involvement**					
Negative (***n*** = 61)	10	16.4	51	83.6	0.08
Positive (***n*** = 33)	9	27.3	24	72.7	
**Tumor size**					
<2 cm (***n*** = 44)	7	15.9	37	84.1	0.22
>2 cm (***n*** = 50)	12	24	38	76	
**Estrogen receptor (ER)**					
Negative (***n*** = 28)	4	14.8	23	85.2	0.27
Positive (***n*** = 67)	15	22.4	52	77.6	
**Progesterone receptor (PR)**					
Negative (***n*** = 46)	7	15.2	39	84.8	0.11
Positive (***n*** = 48)	12	25	36	75	

**Table 2:** Relationship between soluble CrkL fraction in sera from breast cancer patients and pathological parameters.

Age	Stage at Diagnosis			Current Staging	C_CrkL_ in Serum (pg/mL)
	T	N	M		
49	T1b	N1a	M0	1	0
66	T1a	N0	MX	1	3.31
66	T1c	N(i−)	M0	1	6.94
56	T2	N0(i+)	M0	2	0
52	T2a	N0	M0	2	0.67
47	T1c	N1	N/A	2	1.41
75	T2	N1	M0	2	1.85
44	T2	N1	M0	2	1.91
48	T2	N1	M0	2	2.04
76	T2	N0	M0	2	2.09
65	T2	N0	M0	2	2.32
38	T2	N0	M0	2	4.35
59	T1c	N1	MX	2	4.36
50	T2	N0	M0	2	5.03
55	T2	N/A	N/A	2	5.94
69	T2	N1a	N/A	2	6.51
63	T2	N2	M0	3	0
41	T3	N1a	M0	3	0.04
49	T4	N3	M0	3	4.13
47	T2	N2a	MX	3	7.97
48	T2	N3	M0	4	2.92
68	T2	N1	M0	4	3.19
42	TX	NX	M1	4	4.11
49	N/A	N/A	N/A	4	4.78
62	N/A	N/A	N/A	4	5.29
40	T2	N1	M1	4	6.38
63	T2	N1	M0	4	8.71
50	T2	N3a	N/A	4	8.8
67	T3	NX	M1	4	8.86
42	Normal donors				0.83
53					1.03
61					1.38
47					1.72
49					1.86
39					3.50
70					4.00
51					5.09
58					5.15

C_CrkL_: Crk-like protein concentration.

## Supplementary Materials

The following are available online at https://www.mdpi.com/2072-6694/11/7/961/s1, Figure S1: Intensity of CRKL staining in normal breast tissues and breast cancer tissue samples at 4× and 40× magnification.
