# Supplementary material for: Increased Soluble CrkL in Serum of Breast Cancer Patients Is Associated with Advanced Disease
Source: Cancers (Basel). 2019 Jul 9;11(7):961. doi: 10.3390/cancers11070961 (PMC6679112; doi:10.3390/cancers11070961)
Supplement: Supplementary file 1 [file cancers-11-00961-s001.pdf]

Supplementary Materials

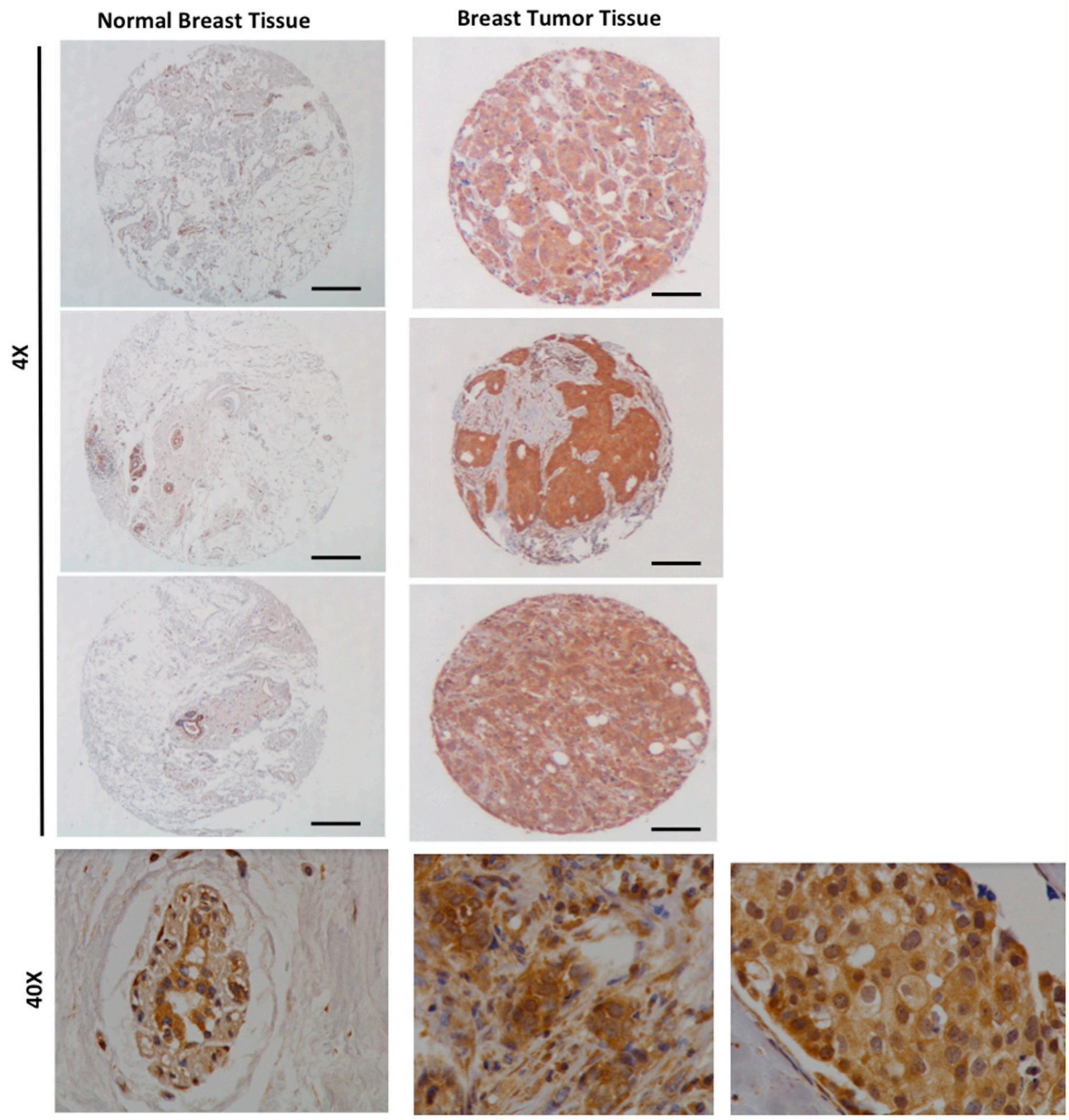

Figure S1. Intensity of CRKL staining in normal breast tissues and breast cancer tissue samples at 4x and 40x magnification. For the normal tissue at 40x magnification only the ductal region which has more intensity is presented. Ductal regions were excluded from the analysis of staining intensity.
